# Influence of the properties of different graphene-based nanomaterials dispersed in polycaprolactone membranes on astrocytic differentiation

**DOI:** 10.1038/s41598-022-17697-9

**Published:** 2022-08-04

**Authors:** Marián Mantecón-Oria, Olga Tapia, Miguel Lafarga, María T. Berciano, Jose M. Munuera, Silvia Villar-Rodil, Juan I. Paredes, María J. Rivero, Nazely Diban, Ane Urtiaga

**Affiliations:** 1 Departamento de Ingenierias Química y Biomolecular, Universidad de Cantabria, Avda. Los Castros s/n, 39005 Santander, Spain; 2 Instituto Marqués de Valdecilla (IDIVAL), 39011 Santander, Spain; 3 Research Group on Food, Nutritional Biochemistry and Health, Universidad Europea del Atlántico, 39011 Santander, Spain; 4 Centro de Investigación Biomédica en Red Sobre Enfermedades Neurodegenerativas (CIBERNED), 28029 Madrid, Spain; 5 Departamento de Anatomía y Biología Celular, Universidad de Cantabria, 39011 Santander, Spain; 6 Departamento de Biología Molecular, Universidad de Cantabria, 39011 Santander, Spain; 7 Instituto de Ciencia y Tecnología del Carbono, INCAR-CSIC, C/Francisco Pintado Fe 26, 33011 Oviedo, Spain

**Keywords:** Biomedical engineering, Biomaterials - cells, Graphene

## Abstract

Composites of polymer and graphene-based nanomaterials (GBNs) combine easy processing onto porous 3D membrane geometries due to the polymer and cellular differentiation stimuli due to GBNs fillers. Aiming to step forward to the clinical application of polymer/GBNs composites, this study performs a systematic and detailed comparative analysis of the influence of the properties of four different GBNs: (i) graphene oxide obtained from graphite chemically processes (GO); (ii) reduced graphene oxide (rGO); (iii) multilayered graphene produced by mechanical exfoliation method (G_mec_); and (iv) low-oxidized graphene via anodic exfoliation (G_anodic_); dispersed in polycaprolactone (PCL) porous membranes to induce astrocytic differentiation. PCL/GBN flat membranes were fabricated by phase inversion technique and broadly characterized in morphology and topography, chemical structure, hydrophilicity, protein adsorption, and electrical properties. Cellular assays with rat C6 glioma cells, as model for cell-specific astrocytes, were performed. Remarkably, low GBN loading (0.67 wt%) caused an important difference in the response of the C6 differentiation among PCL/GBN membranes. PCL/rGO and PCL/GO membranes presented the highest biomolecule markers for astrocyte differentiation. Our results pointed to the chemical structural defects in rGO and GO nanomaterials and the protein adsorption mechanisms as the most plausible cause conferring distinctive properties to PCL/GBN membranes for the promotion of astrocytic differentiation. Overall, our systematic comparative study provides generalizable conclusions and new evidences to discern the role of GBNs features for future research on 3D PCL/graphene composite hollow fiber membranes for in vitro neural models.

## Introduction

The blood–brain barrier (BBB) is a dynamic and complex structure of brain capillaries that selectively control the central nervous system (CNS) homeostasis and protect it from toxins or pathogens^1,2^. Unfortunately, many promising therapeutic strategies did not show the expected results since the BBB represents a critical hurdle for the treatment of CNS diseases. Thus, it prevents most therapeutic drugs from entering the brain due to their physical, biochemical, and specific barrier properties^3,4^.

The development of in vitro neural models constitutes a potentially essential source of knowledge for the understanding of neurodegenerative diseases and the design of novel neuroprotective therapies. They can provide an opportunity for planning new drug-screening platforms, based on neural cell cultures, for cellular trials targeted to neurological disorders. The accomplishment of in vitro BBB models is of utmost importance since they help screening reliably the efficacy of new drugs and therapeutics for brain disorders. The development of dynamic BBB (DIV-BBB) models, which use commercial polymer hollow fibers as platforms for endothelial cells culture in their luminal surface and recreate the physiological environment in terms of fluid dynamics, has demonstrated much more efficient in vitro BBB reconstruction (measured indirectly through the transendothelial electrical resistance (TEER)) than the reference Transwell method^1,5^. However, DIV-BBB models still present much lower TEER values than native BBB tissues.

The co-culture of endothelial cells with astrocytes has been proposed as an efficient procedure to improve BBB reconstruction in vitro, as it has been observed the key role that astrocytes play in the development and function of the brain, and in regulating the phenotype of endothelial cells in BBB models through the regulation of cell–cell communication via soluble factors and direct astrocyte-endothelial cell interactions^6^. Works on DIV-BBB models usually employ co-cultures of endothelial and C6 rat glioma cells induced to astrocytes^7–9^. The biochemical protocols for C6 differentiation are somehow well standardized and thus these works assume satisfactory astrocytic differentiation. However, the commercial polymer hollow fibers used for this application (polypropylene and polyvinylidene difluoride mainly) present poor cell adhesion and are bioinert. Further, in our previous work^10^, we observed that, despite using standard protocols to induce astrocyte differentiation of C6 cells over the surface of polycaprolactone (PCL) polymer hollow fibers, the rate and quality of differentiation was limited in contrast to glass coverslips positive control.

PCL has been widely used as cell culture platform due to its processing versatility (e.g., high solubility in organic solvents and low melting point), mechanical properties, and the capability to generate engineered three-dimensional structures as extracellular matrices depending on the porosity and conformation of the scaffold^12,13^. Furthermore, PCL processed by means of non-solvent induced separation (NIPS) has been studied for its potential as porous scaffolds for hard- and soft-tissue repair such as small caliber blood vessel, neural tissue regeneration, and for the development of vascularized human liver tissue^10,14–16^. In fact, it has obtained the Food and Drug Administration (FDA) approval as surgical implants and drug delivery devices for tissue engineering and regenerative medicine applications^17^. In order to overcome the limited astrocytic differentiation that polymers such as PCL promotes in vitro, numerous approaches have been investigated including blend formation, surface functionalization, and the introduction of fillers^12,13^.

In the last few years, the use of graphene-based nanomaterials (GBNs) in biomedical applications such as drug delivery, diagnostics tests, cancer therapy, and tissue engineering has successfully increased^18,19^. A wide range of nanomaterials such as graphite, mono- or few-layered graphene (FLG), reduced graphene oxide (rGO), graphene oxide (GO), surface functionalized graphene nanomaterials (nitrogen-doped, immobilized with proteins, peptides, etc.), and their derivatives have been considered^20^. They have a common base structure of a single layer of sp^2^ hybridized carbon atoms arranged in a hexagonal lattice, but their chemical and physical properties are different depending on the fabrication methods used (mechanical exfoliation, electrochemical exfoliation, chemical vapor deposition, arc discharge, etc.)^21,22^. Comparative studies of different dispersions of GBN types (FLG, GO and rGO) in contact with astrocyte and neural cell lines proved the effect of these nanomaterials had on the mechanisms of cell differentiation via GBN internalization^23–25^. Other works have studied cell biocompatibility and potential differentiation on 2D film graphene-based substrates^26–30^. Additionally, some investigations have been focused on developing graphene-based composite biomaterials to act as scaffolds for promoting the regeneration of dysfunctional neural tissues, enhancing neural differentiation and neurogenesis^20,31,32^. All these studies have found positive results in both neuronal and non-neuronal cell differentiation with very assorted GBNs such as CVD graphene, rGO, GO, and few-layer GO, among others. Nevertheless, the great experimental and commercial variability of GBNs, and the controversial results about the underlying mechanisms of cellular adhesion, proliferation, and differentiation, remain unclear and require further investigation. In example, certain works clearly report that GO presents better neural differentiation responses^23,33^ than other type of GBNs as FLG or pristine graphene while other works show opposite results^34,35^. Taking all this together, it is justified the need of developing a comparative study to clarify a key question: what properties need to have any GBN to induce neural differentiation?

As a novelty, this work compares the influence of the properties of different GBNs under the same experimental conditions on astrocytic differentiation, using PCL as polymeric matrix, which facilitates the manipulation and subsequent practical use in the development of in vitro dynamic models. For the comparative study, it has been used four types of GBNs: (i) G_mec_, a multilayered graphene produced by mechanical exfoliation; (ii) G_anodic_, a low-oxidized graphene prepared via anodic exfoliation; (iii) GO, produced by chemical oxidation of graphite and exfoliation; and (iv) rGO, produced by hydrothermal reduction of GO. The analysis has been performed for simplicity in flat PCL/graphene-based (PCL/GBN) composite membranes synthetized by phase inversion, which have been characterized to compare the role of the different GBNs on the physicochemical properties and cellular mechanisms involved in cell differentiation. Particularly, some studies have suggested that astrocytes can act as important mediators of the BBB formation by (i) inducing functional barrier properties in brain capillaries (formation of tight junctions), (ii) providing architectural support for neurons, (iii) regulating the exchange of molecules in and out from the brain, (iv) controlling and modulating neurotoxic effects, and (v) maintaining brain homeostasis^2,36^. In consequence, cultured rat C6 glioma cells, widely reported as an experimental model for astrocytic differentiation^37,38^, have been used for cell studies. To determine the degree of astrocytic differentiation, we have analyzed the expression level of cell-specific astrocyte molecular markers. They include genes encoding the glial fibrillary acidic protein (Gfap), a key intermediate filament protein of astrocyte cytoskeleton, and glutamate aspartate transporter (Glast), involved in buffering of synaptically-released glutamate by its removal through uptake into astrocytes^39,40^. Moreover, we have investigated the expression of nestin protein, a component of intermediate filaments enriched in astrocyte protrusions and involved in the morphological plasticity of astrocytes during CNS development and injury^41^.

Therefore, the main aim of this research is to perform a systematic and detailed analysis of the effect of the properties of four GBNs synthetized by distinct procedures on astrocytic differentiation to step forward towards the translation to clinical use.

## Results

### Characterization of graphene-based nanomaterials

**Figure 1:**
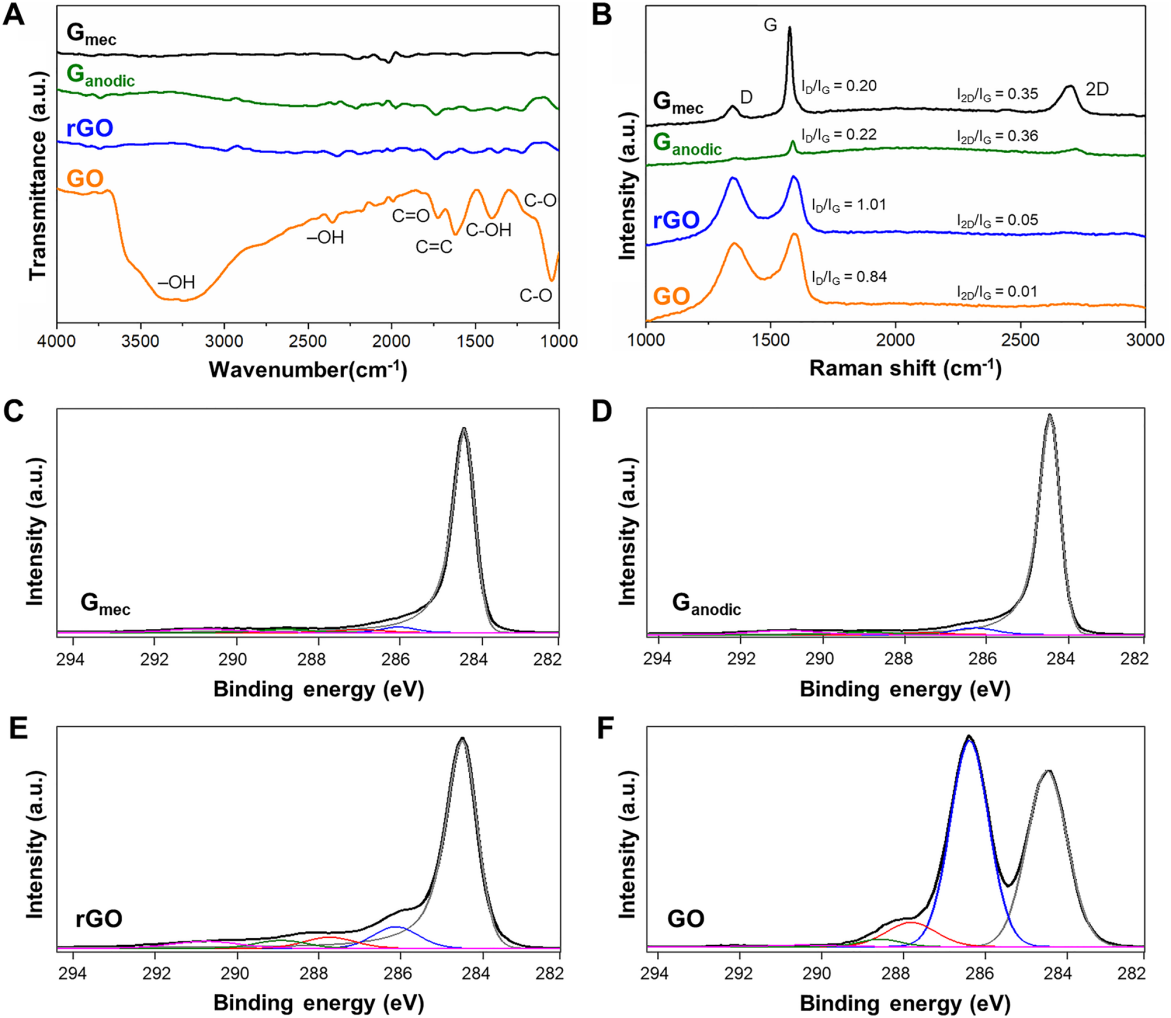
Characterization of G_mec_, G_anodic_, rGO and GO nanomaterials by (**A**) FTIR, and (**B**) Raman spectroscopy. XPS spectra deconvolution of carbon peaks for (**C**) G_mec_, (**D**) G_anodic_, (**E**) rGO, and (**F**) GO. The experimental spectra (black trace) are deconvolved into five components: graphitic carbon band with a maximum at binding energy (BE) ~ 284.5 eV (gray trace); carbon singly bonded to epoxy and hydroxyl groups centered ~ 2 eV higher in BE than the previous component (blue); doubly bonded carbon in carbonyl groups ~ 3.5 eV higher in BE than the graphitic component (red); carbon in carboxyl groups (~ 4.5 eV higher, green); and ${\uppi } \to {\uppi }$* satellite of the graphitic signal (~ 6.5 eV higher, magenta).

Figure 1A exhibits the FTIR spectra of G_mec_, G_anodic_, rGO and GO nanomaterials. GO spectrum displayed the presence of C–O alkoxy stretching vibration^42^ at 1047 cm^−1^, C–O–C epoxy groups at 1220 cm^−1^ (shoulder feature)^43^, C–OH alcoholic at 1405 cm^−1^, skeletal C = C vibration of graphitic domains at 1622 cm^−1^, C=O bonds of carboxylic acid and carbonyl groups at 1726 cm^−1^ and 2359 cm^−1^, respectively, in addition to a broad band, from 3000 to 3700 cm^−1^, corresponding to the hydroxyl O–H group^44,45^. In contrast, in the other GBNs only weak bands of C=C and C=O bonds emerged, revealing the scarcity or even absence of oxygen-containing functional groups.

Figure 1B shows the Raman spectra of the GBNs in absolute arbitrary units with three characteristic bands: D, G and 2D. The D-band is attributed to a breathing mode of A_1g_ symmetry at 1354 cm^−1^, which is associated with the presence of sp^3^ disorder sites or structural defects such as vacancies and edge defects^46,47^. The G-band is a first order scattering of the E_2g_ mode and arises from the C=C stretching vibrations of sp^2^ bonded carbons in graphene planes at 1580 cm^−1^. Finally, the 2D-band, located at 2690 cm^−1^, is a second-order vibration of the D band caused by the scattering of phonons at the zone boundary of the graphite plane^48^. The intensity ratio of the D and G bands (I_D_/I_G_) was calculated^49,50^. For G_mec_ and G_anodic_, I_D_/I_G_ ratios were 0.20 ± 0.07 and 0.22 ± 0.03, respectively. On the contrary, the D-band strongly appeared on GO and rGO nanomaterials because of the high presence of defects, and the I_D_/I_G_ ratio of 0.84 ± 0.06 for GO increased up to 1.01 ± 0.12 for rGO due to the reduction process^47,50^. Additionally, the I_2D_/I_G_ ratio elucidates the transition of sp^2^-hybridized carbon bonds from crystalline nanomaterials to an amorphous state and gives information about the structural quality of the materials^51^. The higher I_2D_/I_G_ ratios of G_mec_ (0.35 ± 0.01) and G_anodic_ (0.36 ± 0.01) in comparison to rGO (0.05 ± 0.03) and GO (0.01 ± 0.01) illustrated the higher structural quality of these nanomaterials. The low I_2D_/I_G_ ratio of rGO and GO GBNs could be related to the breaking of the sp^2^ hybridized lattice by the introduction of oxygen functional groups and the presence of defects, which also explained the shift of G band from 1580 to 1590 cm^−1^ on both GO and rGO^51,52^. Finally, the apparent in-plane crystallite size along the crystallographic a-axis (L_a_) was calculated^53^ with values of 96 nm, 87 nm, 19 nm, and 23 nm for G_mec_, G_anodic_, rGO, and GO nanomaterials, respectively. The low L_a_ values of rGO and GO nanomaterials could be attributed to the presence of oxygen functional groups and related structural disorder (sp^3^ hybridization, vacancy defects, pinholes, etc.) in the graphene lattice^51^.

Figure 1C–F show the deconvolution of C 1 s core level XPS spectra for all GBNs. Likewise, Table 1 collects the different types of bindings between carbon atoms and oxygen atoms. Notably, the deconvoluted curves of G_mec_ (Fig. 1C), G_anodic_ (Fig. 1D), and rGO (Fig. 1E) nanomaterials revealed the limited degree of oxidation of these GBNs, with a large atomic percentage of graphitic C and a high level of electronic conjugation (${\uppi } \to {\uppi }$* satellite band) in these samples (Table 1). In rGO, some epoxy and hydroxyl groups were still in the nanomaterial after the reduction process. Notably, the low presence of aromatic domains in GO (Fig. 1F) is reflected in the low intensity of the ${\uppi } \to {\uppi }$* satellite associated to its graphitic signal (Table 1). XPS survey spectra (Fig. S1) confirmed the presence of C and O in GBNs, with C 1 s and O 1 s core levels at 284.5 eV and ∼ 532 eV, respectively^54^. Table S1 shows the elemental composition of the GBNs with an oxygen atomic percentage of GO (29.4%) > rGO (10.8%) > G_mec_ (3.6%) > G_anodic_ (3.0%). We note that, although in principle, the oxygen content could be also evaluated from the data gathered in Table 1 by making some assumptions on the bond type (e. g., epoxy or hydroxyl), the intrinsic asymmetry of the graphitic carbon band would usually lead to an overestimation of the oxygen atomic percent. Indeed, even when the graphitic bands are fitted with an asymmetric function, as in the present work (see “Graphene-based nanomaterials synthesis and characterization” for details), part of the intensity actually coming from the tail of the graphitic signal is wrongly assigned to oxygen-containing groups. However, although the oxygen content is more accurately determined from the survey spectra (Table S1), the deconvolution data are still useful for a semi-quantitative comparison between the different materials (Table 1).

**Table 1:** Percentage of carbon–oxygen bonds in GBNs calculated from C 1 s XPS deconvoluted spectra.

GBNs	Graphitic C (at%)	C–O (at%)	C=O (at%)	O–C=O (at%)	${\uppi } \to {\uppi }$* (at%)
G_mec_	86.4	3.7	1.8	3.2	4.9
G_anodic_	84.4	5.6	1.8	2.8	5.5
rGO	72.3	11.2	6.5	4.6	5.4
GO	42.6	47.9	7.2	1.8	0.6

We note that although these values are useful for a semi-quantitative comparison between the different materials, the oxygen content is more accurately determined from the survey spectra (data shown in Table S1).

In short, graphene defects are in general related to sp^3^ carbon present in grain boundaries, oxygenated groups or graphitic binding vacancies. G_mec_ and G_anodic_ have high rate of graphitic carbon and low oxygen content, therefore, the low amount of defects that these GBNs present could be mainly attributed to grain boundary defects. GO has large percentage of oxygen groups, which explain the low graphitic carbon present in this material. Meanwhile, rGO has recovered partially the graphitic carbon content and it presents a reduction in the oxygen content in contrast to GO, but Raman data point to similar degree of structural defects than GO. This could be explained by the presence of graphitic binding vacancies in rGO typically attained during the reduction process of GO.

### Characterization of PCL/graphene-based flat membranes

**Figure 2:**
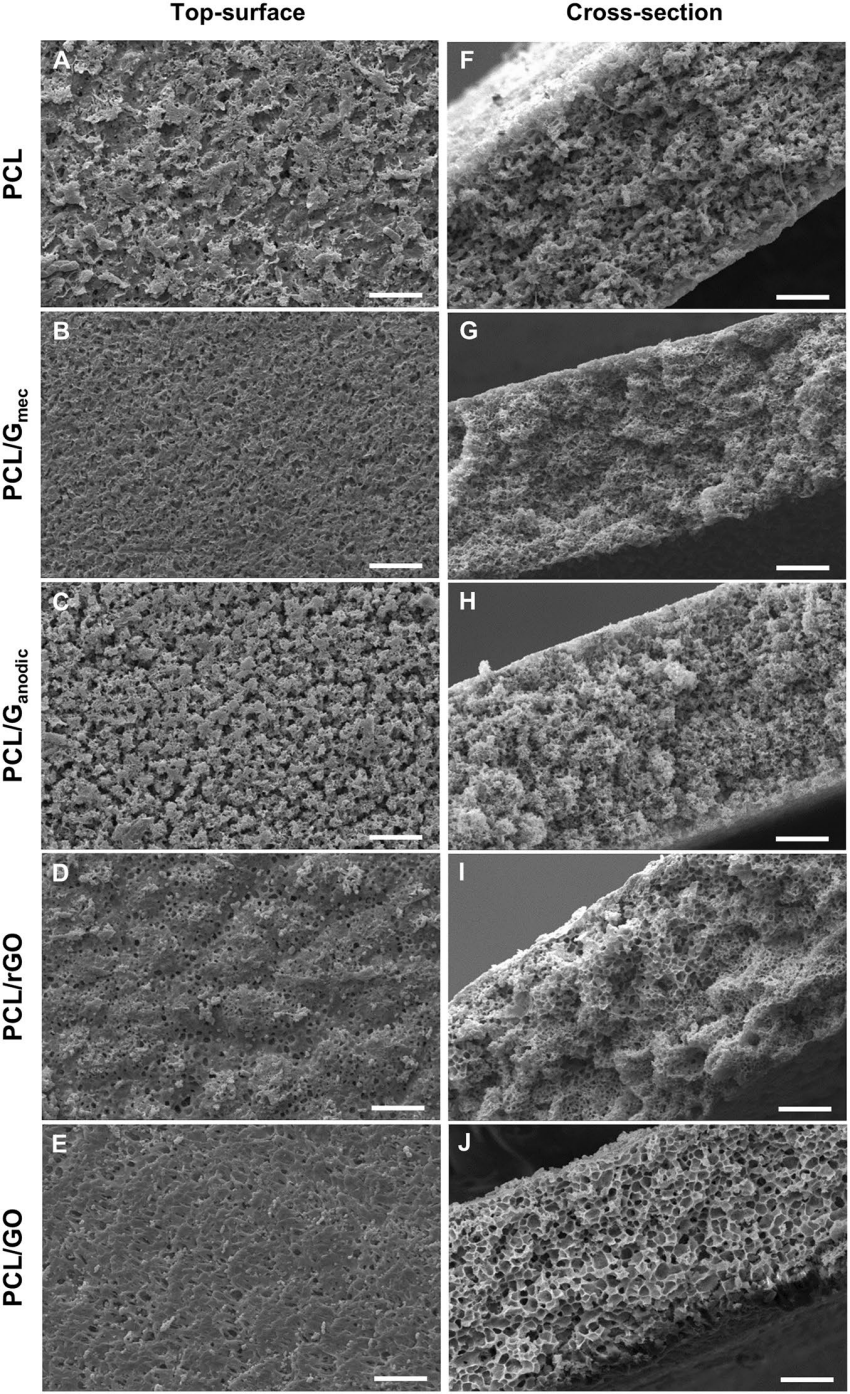
(**A**–**E**) Top-surface and (**F**–**J**) cross-section SEM images of the PCL and PCL/GBN membranes. Scale bar 30 µm.

Figure 2A–E and F–J show SEM images of PCL/GBN membranes of top surface and cross-section morphology, respectively, that exhibit different morphology depending on the GBN incorporated. Table 2 collects the thickness, bulk porosity, surface porosity, and both average surface pore size and cross-section pore size of the membranes.

**Table 2:** Comparison between the morphological parameters of the PCL and PCL/GBN membranes.

Membrane	Thickness (µm)	Bulk porosity (%)	Surface porosity (%)	Avg. surface pore size (µm)	Avg. cross-section pore size (µm)
PCL	130 ± 10	74.5 ± 1.0	31.3 ± 2.2	2.8 ± 0.4	3.6 ± 0.5
PCL/G_mec_	126 ± 5	70.1 ± 1.7	24.7 ± 1.7*	2.4 ± 0.5**	2.4 ± 0.4**
PCL/G_anodic_	136 ± 7	70.5 ± 1.1	34.8 ± 1.3	3.1 ± 0.5**	2.4 ± 0.4**
PCL/rGO	129 ± 6	72.8 ± 1.8	34.8 ± 0.6	3.1 ± 0.5**	3.5 ± 1.4
PCL/GO	121 ± 5	68.3 ± 0.9	30.3 ± 0.4	3.1 ± 0.5**	4.5 ± 0.5**

Statistical significance with respect to PCL (*p < 0.05; **p < 0.01).

PCL/GBNs membranes show similar thickness and bulk porosity without significant statistical difference in comparison to PCL (Table 2). All membranes exhibited an internal sponge-like structure with closed-cellular micropores, characteristic of phase-separated polymer membranes^55^. Moreover, they presented high bulk porosity (~ 70%) and interconnectivity between pores that can potentially provide favorable nutrient transport properties, benefit molecular transfer, and could be suitable for cell adhesion and proliferation. The surface porosity (Fig. 2A–E) remained similar when GBNs were introduced except for PCL/G_mec_ membranes that was reduced from 31.3 ± 2.2% for PCL to 24.7 ± 1.7%. Similar behavior was observed for the average surface pore size. It is noteworthy that the average cross-section pore size of PCL/GO membranes was significantly larger than that of PCL membranes (Table 2). This could be associated with the high O/C atomic ratio of 0.44 in GO, so that the latter might attract molecules of the IPA coagulant via the formation of hydrogen bonds, thus accelerating the phase separation.

**Figure 3:**
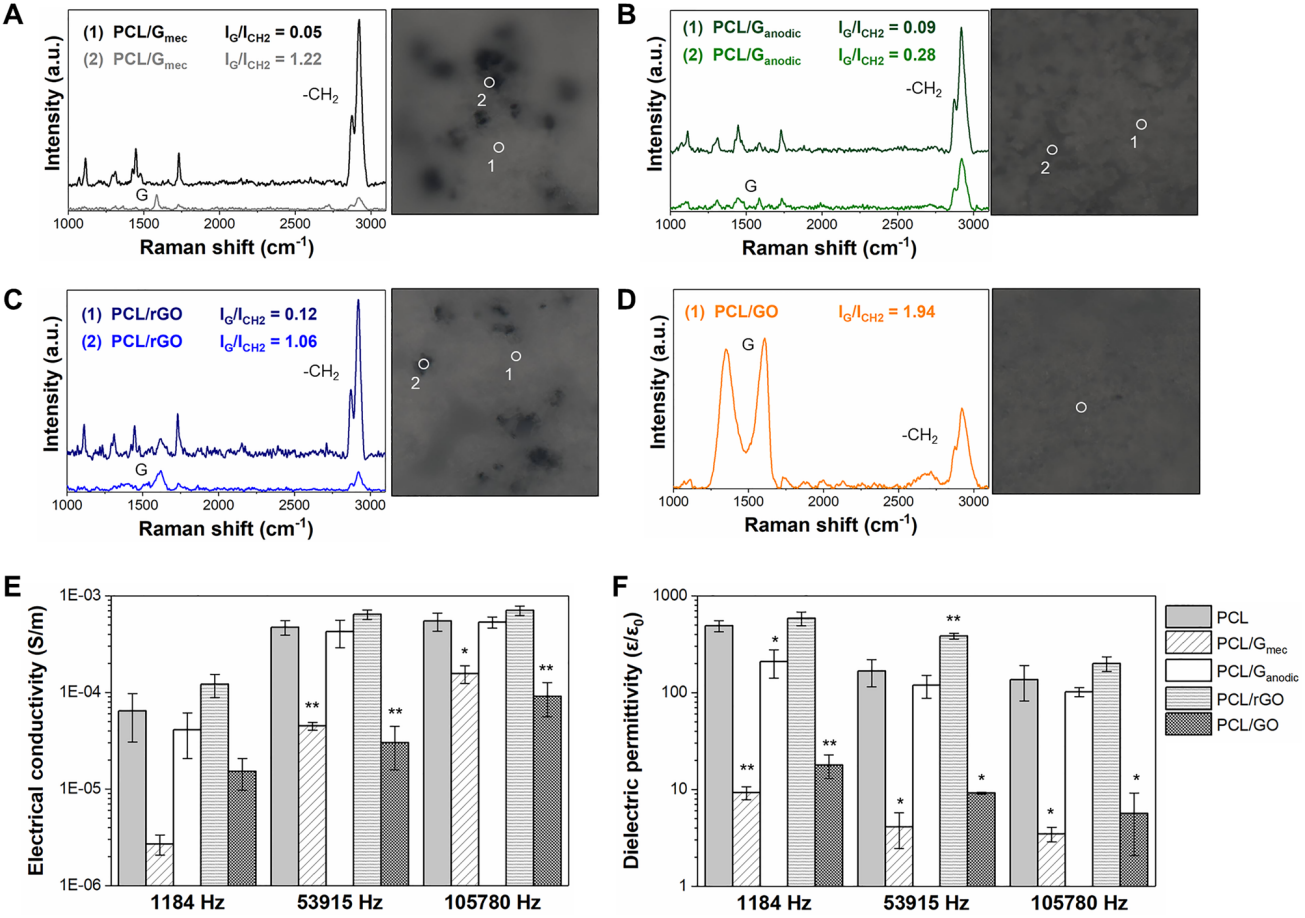
Physicochemical characterization and electrical properties of PCL and PCL/GBN membranes. Raman spectra and optical images of a surface mapping of 100 × 100 µm for (**A**) PCL/G_mec_, (**B**) PCL/G_anodic_, (**C**) PCL/rGO, and (**D**) PCL/GO membranes. The dispersion of GBNs is shown by I_G_/I_CH2_ ratio calculations in each discrete point. Points (1) and (2) exhibit lower and higher content of GBNs, respectively, and ○ represents the analysis zone by the Raman-Confocal laser. (**E**) Electrical conductivity and (**F**) dielectric permittivity properties. Statistical significance with respect to PCL (*p < 0.05; **p < 0.01).

Figure 3A–D depict the Raman spectra and optical images of PCL/GBN surface membranes to evaluate the dispersion of the GBNs. The different I_G_/I_CH2_ ratios meant dissimilar distribution of GBNs throughout the membrane surface with a degree of homogeneity in the dispersion as follows: PCL/GO > PCL/G_anodic_ > PCL/rGO > PCL/G_mec_. Particularly, on PCL/G_mec_ membranes (Fig. 3A) there were areas where G_mec_ was not detected. In contrast, the rest of PCL/GBN membranes presented GBNs concentration at any point measured on the surface. It is also remarkable the low absolute intensity of the Raman spectrum of PCL/G_anodic_ membranes (Fig. 3B) and the presence of high intensity bands corresponding to GO throughout PCL/GO surface (Fig. 3D). Raman and FTIR spectroscopy in Fig. S2A,B, respectively, confirm the presence of characteristic bands of GBNs in the PCL membranes.

Electrical conductivity and dielectric permittivity, Fig. 3E,F, respectively, were evaluated by impedance measurements at 1184 Hz, 53,915 Hz, and 105,780 Hz. It must be considered that under physiological conditions in animal studies^56^, frequencies of at least 1000 Hz are required to induce electrical conduction through neural axons. In general, all membranes exhibited the typical behavior reported for electrical insulators with electrical conductivity increasing (Fig. 3E) and the dielectric permittivity decreasing (Fig. 3F) with frequency. The low electrical conductivity of PCL membranes was comparable to the value reported elsewhere^57,58^, confirming the insulator behavior of this polymer. Specifically, the electrical conductivities found for PCL (5.5·10^–4^ S m^−1^) and PCL/rGO (7.1·10^–4^ S m^−1^) membranes were similar to the values reported previously by Sánchez-González et al.^59^ (9.9·10^–4^ S m^−1^ and 1.8·10^–3^ S m^−1^ for PCL and PCL/rGO membranes, respectively). Therefore, there were no significant improvements in bulk electrical properties when GBNs were introduced into the polymeric matrix since the 0.1 wt% concentration of nanoparticles loaded was insufficient to reach the electrical percolation threshold^13,48^. Nevertheless, a significant decline in the electrical conductivity properties for PCL/G_mec_ and PCL/GO membranes compared to PCL is noteworthy.

**Figure 4:**
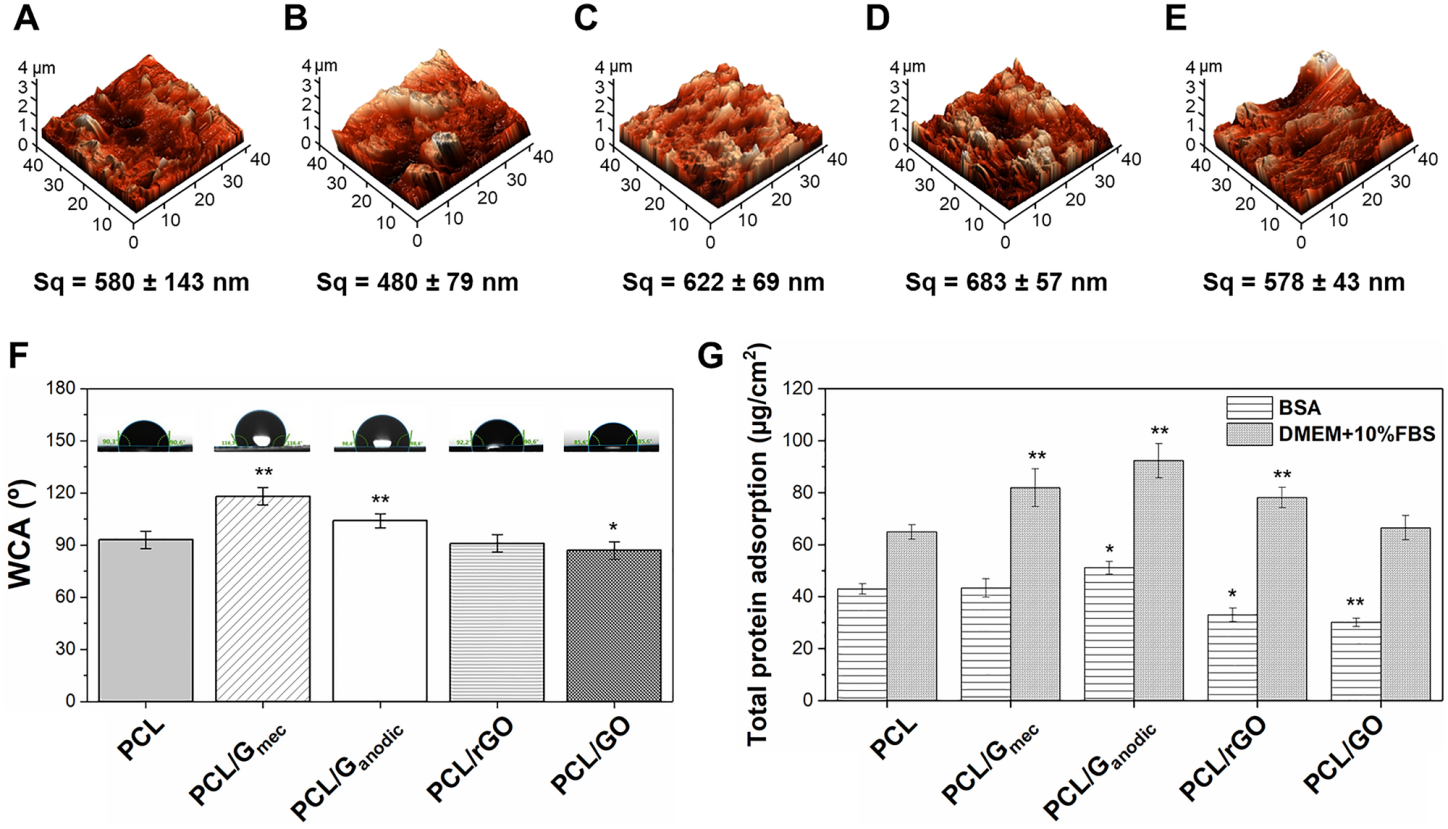
Topographical and protein adsorption characterization of PCL and PCL/GBN membranes. 3D images from 40 × 40 μm scanning areas and surface roughness values by AFM for (**A**) PCL, (**B**) PCL/G_mec_, (**C**) PCL/G_anodic_, (**D**) PCL/rGO, and (**E**) PCL/GO membranes. (**F**) WCA measurements on membrane surfaces. (**G**) Total adsorption of BSA model protein and DMEM + 10% FBS cell culture media on flat membranes. Statistical significance with respect to PCL (*p < 0.05; **p < 0.01).

Surface roughness of PCL and PCL/GBN membranes (Fig. 4A–E) increased in the order: PCL/G_mec_ < PCL/GO < PCL < PCL/G_anodic_ < PCL/rGO, with values showing micro-order topographic roughness and without statistically significant differences among samples. Figure 4F presents water contact angle (WCA) values of 93 ± 5°, 118 ± 5°, 104 ± 4°, 91 ± 5°, and 87 ± 5° for PCL, PCL/G_mec_, PCL/G_anodic_, PCL/rGO, and PCL/GO, respectively. The introduction of G_mec_ and G_anodic_ nanoplatelets significantly enhanced the hydrophobicity of the corresponding composite membrane compared to pure PCL due to the hydrophobic nature of these GBNs^49^. In agreement with other works^47,59^, PCL/rGO membranes did not show any significant change in the WCA compared to PCL. The statistically significant increase of hydrophilicity in PCL/GO membranes could be attributed to the presence of functional groups on GO nanomaterial as well as the preferential location of this nanomaterial at the membrane surface during phase inversion process^59^, as was evidenced by Raman spectroscopy (Fig. 3D).

The adsorption of the individual BSA model protein and DMEM + 10% of FBS mixture was examined on the different membranes (Fig. 4G). PCL/G_anodic_ membranes presented the highest protein adsorption (51 ± 2 µg·cm^−2^ for BSA and 92 ± 7 µg·cm^−2^ for DMEM + 10% FBS) followed by PCL/G_mec_ and PCL/rGO, and significantly higher than PCL membranes. On the other hand, PCL/GO membranes presented the lowest protein adsorption among all PCL/GBN membranes. The protein adsorption capacity on PCL/G_mec_, PCL/G_anodic_, and PCL/rGO membranes can be attributed to a physisorption process controlled by π-π interactions and hydrophobic forces^60^. In PCL/GO membranes the high presence of oxygen groups contributes to the adsorption of proteins^61,62^. Nonetheless, the SEM images of the surface of the membranes (Fig. 2A–E) evidenced a porous surface morphology in PCL/GO membranes (Fig. 2E) with potentially lower effective adsorption area than the rest of membranes. This could produce an artifact in the normalization of mass of protein adsorbed per membrane surface and explain the abnormally low protein adsorption results obtained for PCL/GO membranes.

### Cell studies on PCL/graphene-based flat membranes

**Figure 5:**
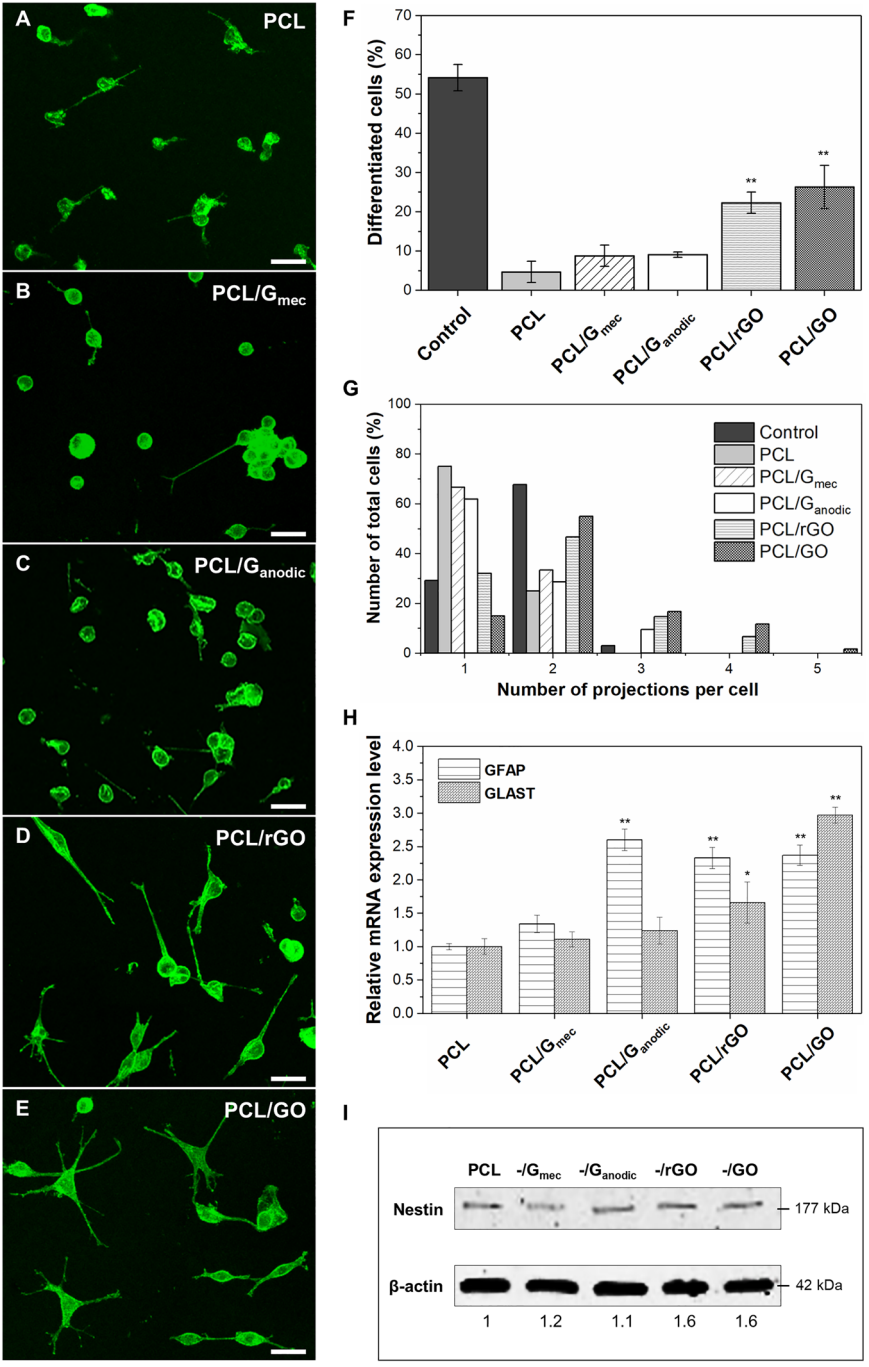
Morphological analysis of C6 cells differentiation towards astrocytes stained with Phalloidin-FITC on (**A**) PCL, (**B**) PCL/G_mec_, (**C**) PCL/G_anodic_, (**D**) PCL/rGO, and (**E**) PCL/GO flat membranes by confocal images. Scale bar = 30 µm. Quantification of (**F**) C6 differentiated cells after 5 days of dbcAMP treatment, and (**G**) number of projections per cell on positive controls and flat membranes. (**H**) Normalized mRNA expressions of Gfap and Glast genes assessed by quantitative real-time PCR, and (**I**) nestin protein expression quantified by Western blotting showing C6 cell differentiation. All Western blot images are presented in Fig. S4. Statistical significance with respect to PCL (**p < 0.01; n ≥ 4).

C6 cell morphology and differentiation towards astrocytes was analyzed in PCL and PCL/GBN membranes (Fig. 5). Figure 5A–E show C6 cells stained with FITC-conjugated phalloidin for F-actin of microfilaments. After 5 days of differentiation, C6 cells exhibited the characteristic reduction of fluorescent actin cytoskeleton signal in all flat membranes. Specifically, Fig. 5D,E showed spindle-shaped morphology in C6 cells with the formation of long thin cytoplasmic projections. Similarly, C6 cells were grown on glass coverslips as positive controls of astrocytic differentiation (Fig. S3). They showed dramatic morphological changes from a flat polygonal shape and well-defined actin cytoskeleton including the characteristic stress fibers of the adhesion stage at day 0 of differentiation (Fig. S3A,B), to a preferential spindle-shaped morphology with long slender cell extensions at day 5 of astrocytic differentiation (Fig. S3C,D). The percentage of C6 cell differentiation is presented in Fig. 5F. A value of 54.2 ± 3.4% was obtained for positive controls whereas for membranes decreased in the following order: PCL/GO (26.3 ± 5.5%) > PCL/rGO (22.3 ± 2.7%) > PCL/G_anodic_ (9.1 ± 0.7%) > PCL/G_mec_ (8.8 ± 2.7%) > PCL (4.7 ± 2.7%). Specifically, PCL, PCL/G_mec_ and PCL/G_anodic_ membranes yielded round-cell morphology (Fig. 5A–C) and 60–75% of the total cells displayed only one cellular projection (Fig. 5G). Remarkably, C6 cells cultured on PCL/G_mec_ (Fig. 5B) formed cell clusters that resembled multicellular spheroid-like structures as previously reported^63^. In contrast, PCL/rGO and PCL/GO membranes substantially stimulated the formation of two to five cell extensions, resembling the typical stellate morphology of astrocytes (Fig. 5D,E,G). Both PCL/rGO and PCL/GO membranes promoted a marked change in cell shape to an asymmetrical shape with elongated processes. Around 22% and 31% of C6 cells exhibited more than two projections on PCL/rGO and PCL/GO membranes, respectively, a proportion significantly higher than that estimated in C6 cells grown on glass coverslips (positive controls), where only 3% of cells had more than two cellular projections.

Furthermore, the changes in the expression of genes encoding two key proteins, Gfap and Glast, for C6 cell differentiation towards astrocytes were analyzed. RT-qPCR analysis of *Gfap* expression in C6 cells grown on the studied membranes showed the following relative mRNA levels: PCL/G_anodic_ (2.60 ± 0.16) > PCL/GO (2.37 ± 0.15) > PCL/rGO (2.33 ± 0.16) > PCL/G_mec_ (1.34 ± 0.13). Interestingly, the expression levels in PCL/G_anodic_, PCL/GO and PCL/rGO membranes were significantly higher in comparison with the level in PCL membranes normalized to 1 (Fig. 5H). Regarding *Slc1a3* (Glast) expression, only C6 cells grown on PCL/rGO (1.66 ± 0.31) and PCL/GO (2.97 ± 0.06) membranes showed significantly increased levels when compared with C6 cells cultured on PCL membranes (Fig. 5H). Finally, Fig. 5I presents the Western blotting of cell lysates, revealing a relative 1.6-times increase in nestin protein expression in C6 cells cultured on PCL/rGO and PCL/GO membranes compared to PCL.

## Discussion

Different GBN characteristics may influence PCL/GBN membrane properties and induce distinct cellular responses and cell-material interactions. Our results indicate that the introduction of a theoretical concentration of 0.67 wt% of rGO and GO nanomaterials into PCL polymeric matrix significantly increased the percentage of differentiated C6 cells, as well as the number of their cellular extensions, in comparison to PCL and other PCL/GBN membranes. Moreover, *Gfap* and *slc1a3* (Glast) mRNAs, and nestin protein expressions increased in both PCL/rGO and PCL/GO membranes. Specifically, Gfap is an astrocyte intermediate-filament protein that plays a key role in both determining cell shape and maintaining the structural support for the spatial astrocytic network of the CNS. In particular, perivascular astrocytes are essential for the establishment of the BBB, including the barrier properties of endothelial cells (tight junctions) and the selective transport in and out of the brain of water and different solutes of the BBB^41,64^. Therefore, the expression of Gfap is required for a successful C6 cell differentiation towards astrocytes. The rise in Gfap expression on PCL/G_anodic_, PCL/GO and PCL/rGO membranes in more than 2.3-fold was comparable to the results obtained in literature for 3D scaffolds^65–67^. In this context, Song et al.^65^ demonstrated that the incorporation of 0.1 wt% of GO on PCL/GO nanofibers, increased 1.7-fold the expression of Gfap in neuron-like rat pheochromocytoma cells (PC12 line) while neural stem cells (NSCs) favorably differentiated into astrocytes on rGO microfibers or graphene foams, enhancing the expression of Gfap by 1.43-fold and 2.5-fold, respectively^66,67^. Additionally, the buffering properties of astrocyte glutamate transporters, such as Glast, keep extracellular brain levels of glutamate at low concentrations since its accumulation in the synaptic space produces neuronal overstimulation and excitotoxicity, and has been involved in several neurodegenerative diseases^64,68^. Several works reported Glast localization in the cell membrane and cellular extensions in cultured rat brain astrocytes^36,64,68^. Interestingly, some authors identified a functional overlap between Gfap and Glast^69,70^. Chiacchiaretta et al.^23^ described that astrocytes actively interact with different GBNs promoting their stellation and increasing Gfap immunoreactivity and glutamate uptake, whereas Sullivan et al.^70^ proposed that Gfap protein allows the retention of Glast transporter via intermediate-linking proteins in the plasma membrane to favor glutamate homeostasis. In this regard, although PCL/G_anodic_ membranes exhibited high capacity to activate Gfap expression in cells (Fig. 5H), the joint participation of Gfap and Glast may be crucial for promoting cytoskeleton reorganization, growth of cell extensions, and glutamate uptake during astrocytic differentiation.

Finally, nestin is one of the intermediate filaments expressed in the earliest stages of brain development, and it is found in cell protrusions as well as at the growing tips of astroglial cell extensions. Nestin can form heteropolymeric filaments with either vimentin or Gfap as obligatory partners. Indeed, it has been stated that Gfap controls nestin network dynamics^41,64^. In this sense, as happened in our work, Guo et al.^66^ also reported the presence of nestin positive cells on rGO microfibers after 3 days of NSCs culture.

**Figure 6:**
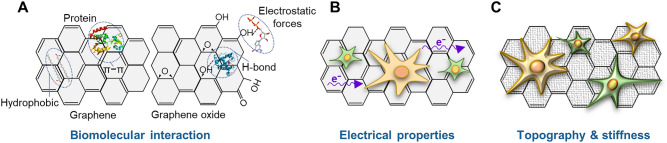
The underlying mechanisms behind the modulation of cellular differentiation due to the presence of GBNs: (**A**) biomolecular interactions, (**B**) electrical properties, and (**C**) surface topography and local stiffness influence [adapted from Luong-Van et al.^52^].

It is important to address the possible cell-material interactions related to the substrate characterizations performed that could explain the promotion and modulation of C6 cells differentiation towards astrocytes thanks to the GBNs addition. Figure 6 presents the feasible GBNs physicochemical properties potentially affecting cellular differentiation processes: (i) biomolecular interactions, (ii) electrical properties, and (iii) topography and local stiffness.

The biomolecular interactions (Fig. 6A) represent the capacity of GBNs to bind proteins, growth factors, and biomolecules through different chemical interactions such as hydrogen bonding, electrostatic forces, hydrophobic interactions, and ${\uppi } - {\uppi }$ bonds. These interactions could increase their local bioavailability, promoting heavy cellular-material interactions and inducing different cellular responses^27,52^. This hypothesis could be confirmed because after 1 day of cell culture, we observed that GBNs highly improved C6 cell attachment and proliferation that could be related to the enhancement of culture medium proteins adsorption (Fig. 4G). Thus, on GBN membranes, protein adsorption was promoted by hydrophobic and ${\uppi } - {\uppi }$ interactions associated with the extended electronically conjugated (aromatic) domains present in G_mec_, G_anodic_, and rGO, as revealed by XPS (Table 1). By contrast, such domains were less abundant in GO, thus pointing to protein adsorption levels that were comparable to those of PCL. Additionally, the surface morphology of PCL/G_anodic_ membrane (Fig. 2C) presented cavities that may act as molecular reservoirs, leading to a higher effective specific surface area than in a flat surface area and thus resulting in increased protein adsorption. For BSA protein, strong interaction with hydrophobic biomaterials was previously attributed to protein-substrate intermolecular binding via hydrophobic patches and amino acid chains^27,28,32^. Moreover, the significantly lower BSA adsorption on PCL/rGO and PCL/GO membranes was justified by the presence of electrostatic repulsions between the negatively charged protein at pH 7.4^71^ and the oxygen groups confirmed by a content of oxygen of 10.8 at% and 29.5 at% for rGO and GO, respectively (Table S1). Presumably, this electrostatic repulsion reduced the adsorption of BSA on PCL/rGO and PCL/GO membrane surfaces and may produce a higher adsorption of other proteins such as fibronectin, laminin or vitronectin, which are three components of the cellular matrix and culture media, playing an important role in the cellular attachment to the substrate, the modulation of NSCs behavior, and neuronal outgrowth and migration^61,72^. Remarkably, laminin protein interacts with and controls membrane proteins, such as lamin-B receptor, enhancing the linkage of the extracellular matrix of the cell membrane to chromatin, and promoting cell differentiation^73,74^. Specifically, it was shown that laminins act as haptotactic factors in vitro, i.e., inducing astrocyte migration and promotes its differentiation^75^. Therefore, the lower adsorption of BSA globular protein on PCL/rGO and PCL/GO may enhance the adsorption of laminin protein, and more importantly, the promotion of a direct linkage between the cellular receptors to the membrane surface, triggering cell differentiation.

Focusing on wettability, the hydrophilic character of surface substrates can lead to increased cell anchorage by improving the degree of contact between cellular receptors and the physiologic environment^76^. PCL/GO membranes were slightly hydrophilic with a WCA of 87 ± 5°, whereas the other membranes were more hydrophobic, with average values higher than 90°. The interaction between the water droplet and the surface of PCL/GO membranes decreased the WCA for these membranes due to the presence of oxygen-containing groups. Moreover, their electrostatic interactions could improve physicochemical linkages between cells and surfaces as happened in other works^28,77^.

Another critical point is the existence of structural and chemical defects on GBNs in the form of highly reactive sites or “hot spots” for protein and cell-material interactions^28,52,61^. The chemical oxidation and exfoliation of graphite for GO production, and then its hydrothermal reduction to obtain rGO, produced structural and chemical defects altering the sp^2^ carbon hybridization and distorting the benzene ring structure, with average values of 0.84 and 1.01 for I_D_/I_G_ ratios, respectively. In contrast, G_mec_ and G_anodic_ nanomaterials presented less defects, as was expected due to their synthesis method. Besides, the lowest I_2D_/I_G_ values previously obtained for rGO and GO nanomaterials support the evidence of the highest amount of structural disorder for these nanomaterials since 2D band tends to decrease in the presence of large amounts of disorder^50^. The high presence of vacancy and edge defects, and structural disorder in rGO and GO GBNs could provide highly reactive sites and larger indentation depths to enhance not only protein adsorption but also cell-material interactions and anchorage, which probably influence the cell differentiation processes. Therefore, the presence of defects on GBNs probably provides direct contact between the receptors of the cellular membrane and the membrane surface triggering intracellular cascades that would modulate the differentiation response, and potentially influence cell fate.

The second mechanism involves the critical role of the electrical properties in neural tissues and cell differentiation where cell communication relies heavily on electrical signals as is represented in Fig. 6B. Several works reported that electrically conductive environments could locally modulate biological activity and lead to better cellular differentiation, like neurite outgrowth and sprouting^20,52,76^. In this way, Sánchez-González et al.^59^ suggested that the highest neural activity of matured neural progenitor cells grown on PCL/rGO membranes could be attributed to their higher electrical properties compared to PCL and PCL/GO membranes. Therefore, the electroconductive character of the scaffolds was evaluated. All membranes exhibited insulating behavior (Fig. 3E,F), although a small reduction of electrical properties was observed in PCL/G_mec_ and PCL/GO ones. For PCL/G_mec_, it was attributed to the mechanically exfoliated G_mec_ nature, and the formation of large nanoplatelets aggregates with non-conductive pathways (Fig. 3A), which handicapped charge transport networks as already proposed by Xia et al.^78^ for graphene-polymer nanocomposites. For PCL/GO membranes, the large pores found in the cross-section (Fig. 2J) could produce high resistivity as a result of the larger distances between the conductive nanoparticles^79^. Moreover, GO nanomaterial is known as a poor conductor^65^, and presented 29.4% of oxygen attributed to oxygenated groups (Table S1) that disturb the extended sp^2^-hybridized carbon structure. Overall, we could not find evidence that the electrical properties contributed to cell differentiation response.

Finally, it has been proven previously that surface topography and stiffness play a fundamental role in the contact between cell membrane and substrates (Fig. 6C). These biophysical cues can promote different cell anchorage and mechanosensitive pathways, which produce cellular cytoskeleton rearrangement and trigger a cascade of transduction signals that modulate cell differentiation^28,52^. Although Lee et al.^80^ observed that nanopatterns on rGO and GO films affected neurite outgrowth, our PCL/GBN membranes exhibited similar surface roughness at the microscopic level without statistical significance. Due to the micro-level topography of the membranes, AFM-based analysis of the surface mechanical stiffness was out of the range of sensitivity of the equipment used in this study, and other macroscopic indentation techniques had to be also discarded because they damaged the polymer material. On the other hand, local stiffness strongly influences cell fate initiating mechanotransduction cascades and cell differentiation, and could be affected by the aggregation/agglomeration of GBNs nanoparticles and the presence of chemical structural defects^52,81^. The homogeneous dispersion of GBNs in membrane matrices reinforces their mechanical properties^81^. Both PCL/G_mec_ (Fig. 3A) and PCL/rGO (Fig. 3C) presented agglomerates on membrane surface, whereas the rGO was present throughout the whole PCL/rGO surface, in PCL/G_mec_ membranes there were regions where only PCL was detected. For PCL/G_anodic_ (Fig. 3B) and PCL/GO (Fig. 3D), graphene-based nanoparticles were well dispersed. Furthermore, the incorporation of rGO and GO defective GBNs into the PCL polymeric matrix created topographies in the nanoscale order that, when coupled with the presence of edge or vacancy defects, may produce mechanical stress at cellular level promoting the differentiation^52^. Nevertheless, PCL/GBN membranes contained only 0.67 wt% of GBNs in the solid membrane, and therefore, mechanical stiffness on membrane surface is not expected to change substantially at bulk level.

## Conclusions

In this study, PCL/graphene-based flat membranes were fabricated by phase inversion technique to elucidate if there are optimal features of graphene-based nanomaterials (GBNs) to be dispersed in the PCL polymeric matrix to induce astrocytic differentiation. A concentration of 0.67 wt% of the different GBNs synthetized by distinct methods (G_mec_, G_anodic_, rGO, and GO) was loaded into the PCL membranes. The physicochemical, electrical, and topographical properties of the composite scaffolds were evaluated as well as the protein adsorption capacity and biological functionality. Overall, the characteristic change in C6 cells towards a differentiated, astrocytic morphology coupled with the high Gfap and Glast gene expressions, and the presence of nestin-positive cells, revealed that both PCL/rGO and PCL/GO membranes acted as the most favorable substrates for the enhancement of astrocytic differentiation. These results could be justified by: (i) the presence of chemical structural defects in rGO and GO nanomaterials, and (ii) the selective (i.e. laminin over BSA) protein adsorption capacity of PCL/rGO and PCL/GO membranes. Both mechanisms, favored the interaction of the receptors of the cell membrane with the adsorbed biomolecules on PCL/rGO and PCL/GO substrates, and the direct contact with these flat membrane surfaces. The results confirmed that astrocytic differentiation is modulated by the presence of defects, so GBNs with these features should be selected over crystalline ones. Additionally, the uniform dispersion of GO and rGO nanomaterials throughout the membrane surface favored cell anchorage to accessible and strategic points of the cellular membrane to these PCL/GBN surfaces, and the outgrowth and sprouting of cellular extensions. It is noteworthy that these biocompatible substrates have the potential to induce astrocytic differentiation incorporating only 0.67 wt% of GBNs in the polymeric matrix, not increasing substantially the cost of the membranes and reducing the potential controversy with cytotoxic effects.

As a major novelty, this work systematically compared the influence of the properties of four different GBNs on astrocytic differentiation under equal experimental conditions and scattered into the same PCL polymeric matrix platform allowing to draw generalizable conclusions for future investigations towards the clinical translation of these composite materials.

## Methods

### Graphene-based nanomaterials synthesis and characterization

Different qualities of GBNs were used in this work: graphene/graphite nanoplatelets mechanically exfoliated (G_mec_, Av-PLAT-7, acquired from Avanzare Innovación Tecnológica); graphene with minimal oxidation produced by aqueous anodic exfoliation of graphite with Na_2_SO_4_ as the main electrolyte and NaCl as the co-electrolyte, as we have reported previously^49^ (G_anodic_); graphene oxide nanosheets dispersed in distilled water at 4 mg·mL^−1^ (GO, Graphenea, S.A.); and rGO obtained via a hydrothermal reduction method^82^ from the commercial GO (Graphenea).

The graphene-based nanomaterials were characterized by Fourier-transform infrared (FTIR), Raman, and X-ray photoelectron spectroscopy (XPS). FTIR spectra were obtained using a spectrophotometer (Spectrum 65 Two spectrometer, PerkinElmer, Spain) and operating in the attenuated total reflectance mode (GladiATR, PIKE Technologies, USA). Raman spectra were acquired with a Confocal-Raman NRS-4500 instrument (Jasco, Spain). A 532 nm excitation wavelength was focused with a 50 × MPlan FLN 0.8 NA objective at an effective laser power of 4.4 mW. The data were collected using Spectra Manager™ software. XPS was carried out on a SPECS system under a pressure of 10^–7^ Pa using a non-monochromatic Al K_α_ X-ray source (14 kV at 175 W). A pellet of each GBN powder was prepared for the measurements. XPS data were analyzed using CasaXPS software. For deconvolution of the C 1 s high resolution spectra, the bands were fitted as a (symmetrical) convolution of a Gaussian and a Lorentzian function (80:20), while the graphitic band was fitted with the same convolution modified by a function to approximate its asymmetrical shape, specifically, the asymmetric form due to Ulrik Gelius as implemented in CasaXPS software. Both Raman and XPS data were further processed by Origin Pro 2017 software.

### PCL/graphene-based flat membrane preparation and characterization

PCL/graphene-based flat membranes were prepared by phase inversion adapting the procedure described elsewhere^82^. The nanomaterial concentration was selected based on previous results which confirmed that membranes prepared from solutions with GBNs loading above 0.1 wt% were difficult to handle and mechanically unstable^83^. Furthermore, the use of low quantity of GBNs in the polymer matrix is intrinsically a benefit of this technology as in the one hand it minimizes the potential cytotoxic exposure of the cells to graphene nanomaterials in case they are leached out of the PCL matrix, and in the other hand the production costs associated to graphene are reduced. Therefore, 0.1% w/w of GBNs was homogeneously dispersed by ultrasonication in the solvent N-methyl-2-pyrrolidone (NMP, 99% extrapure, Acros Organics). The different times of ultrasonication to achieve 24 h stable graphene liquid solutions were 40 min for G_mec_ and GO nanomaterials, 70 min for previously exfoliated G_anodic_, and 90 min for rGO. Afterwards, 15% w/w of PCL polymer (MW, 80 kDa, Sigma Aldrich) was added to the GBNs/NMP dispersions and stirred for 48 h at 37 °C until achieving a uniform solution. Polymeric solutions were cast on a glass plate using a 200 µm thickness for the casting knife (Elcometer 3580/2, USA), submerged into a coagulation bath of 100% v/v 2-propanol (IPA, 99%, Oppac), and washed with ultrapure water to remove solvent traces. Finally, the synthetized membranes contained a theoretical concentration of 0.67 wt% of GBNs. For comparison, control membranes containing only PCL (15%w/w PCL in NMP) were also prepared.

The morphology and structure of the cross section and surface of the PCL/GBN flat membranes were determined using scanning electron microscopy (SEM, EVO MA 15, Carl Zeiss, Germany) at a voltage of 15 kV. For cross-section images, membrane samples were frozen in liquid nitrogen and fractured. All the samples were kept overnight at 30 °C under vacuum (200 mbar) and were gold sputtered before examination. To study the surface porosity (n ≥ 3) and the average pore size (n ≥ 100), ImageJ software was used. To determine the thickness (δ) of the PCL/GBN scaffolds, membrane samples (n ≥ 8) were measured using an electronic micrometer (Series 293, Mitutoyo, Spain). In addition, bulk porosity ($\varepsilon$) was measured using a MS-TS hydrostatic weighing system (Mettler Toledo, Spain) and calculated according to Eq. (1), where ${\uprho }_{{\text{m}}}$ is the measured density of the membranes (n ≥ 3) and ${\uprho }_{{\text{t}}}$ is the theoretical density of the PCL polymer.

1 $${\varepsilon }\left( {\text{\% }} \right) = \left( {1 - \frac{{{\uprho }_{m} }}{{{\uprho }_{{\text{t}}} }}} \right)\cdot100{ }$$

The electrical properties of the PCL/GBN membranes were evaluated via impedance spectroscopy with an electrochemical workstation (Zennium, Zahner, Germany) using a direct contact method. The dry membrane samples (n $\ge$ 3) with a specific area of 1.3 cm^2^ were placed between the electrodes in an external holder. All impedance measurements were performed at room temperature in potentiostatic mode, and the frequency varied in the range of 1–600 kHz.

Surface topography was analyzed by atomic force microscopy (AFM, NTegra NT-MDT, Russia) in semi-contact mode using silicon probe NSG-03 and golden silicon cantilevers with resonance frequencies from 47 to 150 kHz and characterized by root mean square surface roughness parameter (Sq). Further, the wettability of the membrane surface (n $\ge$ 11) was evaluated with ultrapure water according to a sessile-drop method by a drop shape analyzer (DSA25, KRÜSS Scientific, Germany). In the evaluation of protein adsorption, 2.5 cm^2^ of PCL/GBN membranes were exposed to two solutions: 10% of fetal bovine serum (FBS, Gibco™) in Dulbecco’s Modified Eagle Media (DMEM, Gibco™) and, 4 g·L^−1^ of bovine serum albumin (BSA, A9647, p ≥ 98%, Sigma Aldrich) in phosphate buffer solution (PBS, pH 7.4). After 24 h of incubation at 37ºC and 5% of CO_2_, membrane samples were rinsed twice with PBS and adsorbed proteins were removed by 1% of sodium dodecyl sulfate (SDS). The total protein was quantified following the bicinchoninic acid protein assay kit (BCA, Pierce™) protocol and using a Spark® microplate reader (Tecan, Switzerland). FTIR and Raman spectra were also recorded on PCL/GBN membranes.

### Cell studies on PCL/graphene-based flat membranes

#### Cell cultures

Membrane samples of 14 mm diameter were glued to glass coverslips to prevent floating, sterilized with 70% v/v ethanol (EtOH, > 99.8%, Honeywell) solutions and UV light, and washed with sterile PBS to remove EtOH traces, all in a laminar flow cabinet.

Cell biocompatibility and astrocytic differentiation on PCL and PCL/GBN membranes were carried out with C6 rat glioma cells (CCL-107™, ATCC®). C6 cells were cultured in DMEM, enriched with 10% FBS, 1% of non-essential amino acids (NEAA) and 1% of penicillin/streptomycin (P/S) at 37ºC in a humid atmosphere containing 5% CO_2_. All supplements were acquired from Gibco™. Culture medium was changed every 2 days until 80% of cell confluence. Then, C6 cells were uniformly seeded (3.2 × 10^4^ cells per cm^2^) on flat membranes. After 24 h, astrocytic differentiation was induced for 5 days using 0.5% FBS in DMEM containing 1 mM of dibutyryl-cAMP (dbcAMP, Sigma Aldrich). Results were compared with glass coverslips as positive control.

#### Cell characterization

Cells cultured on flat membranes were fixed with fresh 3.7% paraformaldehyde in PBS for 15 min, permeabilized with Triton X-100 (0.5% in PBS) for 5 min and washed with PBS. Next, the samples were incubated for 30 min in Phalloidin-FITC conjugate (Sigma Aldrich), mounted on slides with antifade medium Vectashield (VectorLabs) conjugate with DAPI (4´, 6-diamidino-2-phenylindole), and examined under a LSM510 (Zeiss) laser scanning microscope using a 40 × and 63 × oil (1.4 NA) objective.

C6 cell differentiation was evaluated on PCL and PCL/GBN membranes by performing a morphometric analysis of the size and shape of cells in each confocal image (n $\ge$ 4) with ImageJ software, and by RT-qPCR and Western blotting techniques. Both RT-qPCR and Western blotting results were compared with cell cultures on TCPs (Tissue Culture Plastics, positive control).

For RT-qPCR studies, total RNA was extracted individually from C6 glioma cells in each experimental group by Trizol reagent (Invitrogen) and purified with the RNeasy kit (Qiagen). The concentrations of total RNA were determined using a NanoDrop ND-1000 spectrophotometer (Nanodrop Technologies, Spain). 150 ng of purified RNA was reverse-transcribed to first-strand cDNA by a High-Capacity cDNA Reverse Transcription Kit (Life Technologies) using random hexamers as primers. The expression of *Gfap* and *Glast* mRNAs was determined by RT-qPCR using gene-specific SYBR Green-based primers (Invitrogen). Each RT-qPCR assay was carried out in triplicates. The threshold cycle (Ct) for each well was determined and the results were normalized to Gapdh as a reference. Relative gene expression was calculated according to the 2^−(ΔΔCt)^ equation. The DNA sequences of the primers used in this study were as follows: *Gfap* forward 5ʹ-CCAAACTGGCTGACGTTTACC-3ʹ, *Gfap* reverse 5ʹ-TGGTTTCATCTTGGAGCTTCTG -3ʹ, *Glast* forward 5ʹ- GGCTGCGGGCATTCCTC-3ʹ, *Glast* reverse 5ʹ-CGGAGACGATCCAAGAACCA-3ʹ, *Gapdh* forward 5ʹ-CATCAAGAAGGTGGTGAAGCAGG-3ʹ and *Gapdh* reverse 5ʹ-CCACCACCCTGTTGCTGTAGCCA-3ʹ.

For Western blotting, C6 cells cultured for 5 days were scratched off from PCL/GBN membranes and TCP and resuspended in 1 × Laemmli buffer (2% SDS, 0.1% 2-mercaptoethanol, 10% glycerol, 0.0005% bromophenol blue, and 63 mM Tris–HCl pH 6.8) supplemented with complete protease inhibitor cocktail. The samples were boiled for 10 min at 100 °C and cleared by centrifugation at 11,000 rpm for 5 min at 4 °C. Equal amounts of proteins were subjected to 4–20% Nu-Page TG polyacrylamide SDS-PAGE gels (Invitrogen) and transferred to nitrocellulose membranes (0.2 µm pore size, Life Technologies). Then, membranes were rinsed twice with 0.1% Tween-20 (PBS-T) and blocked with 5% non-fat dried milk (Biorad) in TBS/Tw. Finally, membranes were incubated with rabbit polyclonal anti-nestin (Sigma Aldrich, dilution 1:5000 in PBS-T), and goat polyclonal anti-β-actin (Abcam, dilution 1:1000 in PBS-T) primary antibodies and kept shaking at 4 ºC overnight. Afterwards, membranes were incubated for 1 h with specific anti-rabbit and anti-goat secondary antibodies (Rockland Immunochemicals, dilution 1:5000 in PBS-T) at room temperature. Protein bands were detected with an Odyssey™ Infrared-Imaging System (Li-Cor Biosciences, USA), and quantified using ImageJ software.

### Statistical analysis

All results were expressed as mean ± standard deviation. For all characterizations, a one-way analysis of variance (ANOVA) was performed to determine whether there were significant differences between PCL membranes, taken as reference, and the PCL/GBN membranes. The statistical significance level was defined at p $<$ 0.05.

## Supplementary Information

Supplementary Information.

## Data Availability

The RT-PCR gene expression datasets generated and analysed during the current study are available in the Zenodo repository (https://doi.org/10.5281/zenodo.6874249).
