# Supplementary material for: Influence of the properties of different graphene-based nanomaterials dispersed in polycaprolactone membranes on astrocytic differentiation
Source: Sci Rep. 2022 Aug 4;12:13408. doi: 10.1038/s41598-022-17697-9 (PMC9352708; doi:10.1038/s41598-022-17697-9)
Supplement: Supplementary file 1 — Supplementary Information. [file 41598_2022_17697_MOESM1_ESM.pdf]

Influence of the properties of different graphene-based nanomaterials dispersed in polycaprolactone membranes on astrocytic differentiation

Marián Mantecón-Oria[1,2], Olga Tapia[3,4], Miguel Lafarga[2,4,5], María T. Berciano[2,4,6], Jose M. Munuera[7], Silvia Villar-Rodil[7], Juan I. Paredes[7], María J. Rivero[1], Nazely Diban[1,2,*] & Ane Urtiaga[1,2]

[1]Departamento de Ingenierias Química y Biomolecular, Universidad de Cantabria, Avda. Los Castros s/n, 39005 Santander, Spain

[2]Instituto Marqués de Valdecilla (IDIVAL), 39011 Santander, Spain

[3]Research Group on Food, Nutritional Biochemistry and Health, Universidad Europea del Atlántico, 39011 Santander, Spain

[4]Centro de Investigación Biomédica en Red sobre Enfermedades Neurodegenerativas (CIBERNED), 28029 Madrid, Spain

[5]Departamento de Anatomía y Biología Celular, Universidad de Cantabria, 39011 Santander, Spain

[6]Departamento de Biología Molecular, Universidad de Cantabria, 39011 Santander, Spain

[7]Instituto de Ciencia y Tecnología del Carbono, INCAR-CSIC, C/Francisco Pintado Fe 26, 33011 Oviedo, Spain

[*]Corresponding author: dibann@unican.es

Supporting Figures

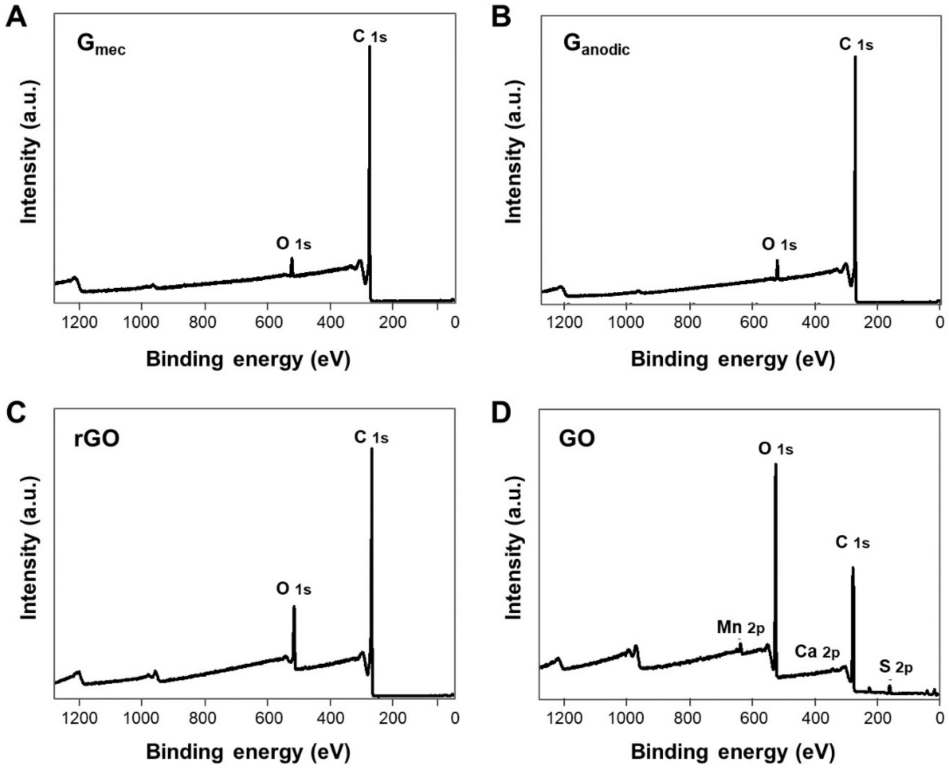

Figure S1. XPS survey spectra for (A) G_{mec}, (B) G_{anodic}, (C) rGO, and (D) GO. The peaks of S, Mn, and Ca on GO nanomaterial derived from the preparation of the sample.

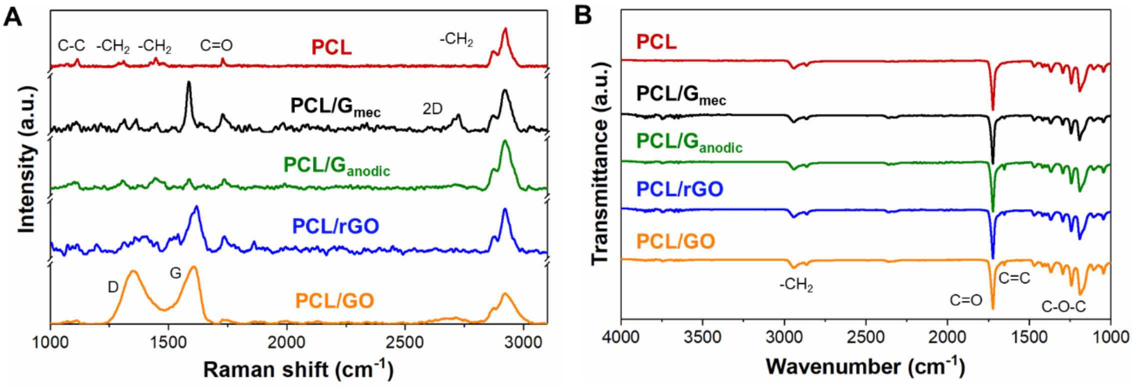

Figure S2. PCL and PCL/GBN membranes physicochemical characterization by (A) Raman, and (B) FTIR spectroscopy.

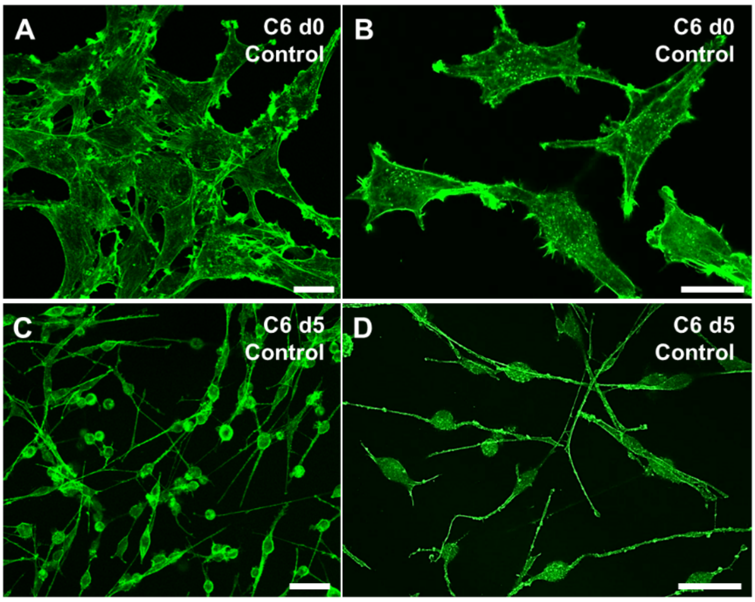

Figure S3. Confocal images showing C6 cellular differentiation process towards astrocytes grown on glass coverslips as positive controls. Morphology of C6 cells in the (A, B) adhesion stage at day 0 with a scale bar of 20 μm, and (C, D) differentiation stage at day 5 with a scale bar of 50 μm.

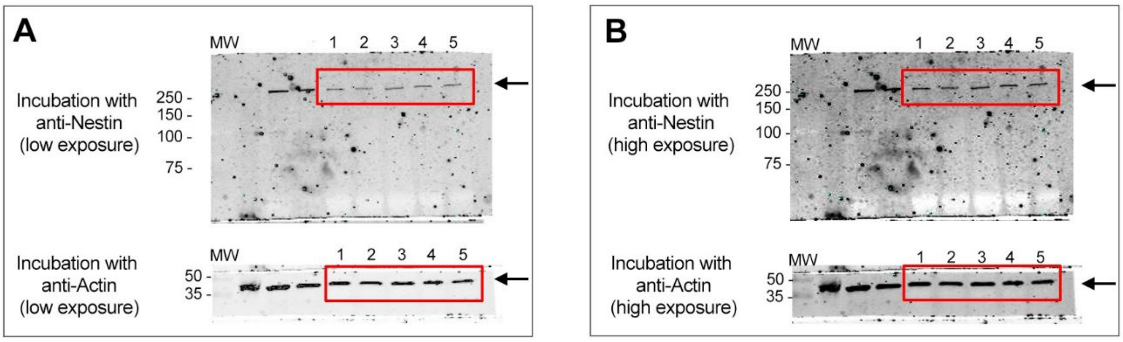

Figure S4. Uncropped blots corresponding to Figure 5I at (A) low exposure, and (B) high exposure. Molecular weights are annotated with labels in the figure and the red box indicates the cropped area shown in Figure 5I.

Supporting Tables

Table S1. Surface composition and O/C ratio calculated from XPS survey spectra.

**GBNs**	**C** (at%)	**O** (at%)	**S** (at%)	**Mn** (at%)	**Ca** (at%)	**O/C** (at%)
G_{mec}	96.4	3.6	-	-	-	0.04
G_{anodic}	97.0	3.0	-	-	-	0.03
rGO	89.2	10.8	-	-	-	0.12
GO	66.5	29.4	2.9	0.9	0.3	0.44

Supporting Information

Raman results (Figure S2A) of PCL showed its characteristic bands: crystalline bonds of C-COO at 1113 cm^{-1}, C=O at 1734 cm^{-1}, and CH_2 symmetric and asymmetric stretching bands at 2896 cm^{-1} and 2925 cm^{-1}, respectively, as well as other CH_2 band regions around 1291-1313 cm^{-1} and 1430-1488 cm^{-1}. Analyzing composite membranes, all presented the bands of PCL polymer and the characteristic G-band confirming the presence of GBNs into the polymeric matrix. Some changes in C=O band and a shift in the vibrational G band from 1580 cm^{-1} to 1610 cm^{-1} confirmed GBNs and PCL polymer interactions, especially in PCL/rGO and PCL/GO membranes [1,2].

FTIR (Figure S2B) of PCL membranes exhibited two strong bands at 1189 cm^{-1}, corresponding to stretching vibrations of the C-O-C ether groups, stretching vibrations of the C=O carboxyl at 1726 cm^{-1}, and other characteristic bands between 1470 to 1045 cm^{-1} representing C–O, C–O–C, C–C and –CH_2 stretching vibrations in the crystalline and amorphous phase of PCL. Moreover, it can be seen the asymmetric and symmetric –CH_2 stretching at 2942 cm^{-1} and 2864 cm^{-1}, respectively. For composite membranes, FTIR bands were slightly displaced probably because of the molecule arrangement and residual tensile stress

between the polymer chain and the GBNs, showing at 1645 cm^{-1} the covalent C=C bond corresponding to the skeletal vibrations from unoxidized graphitic domains [3,4].

References

[1] J. Ahmed, T.A. Tabish, S. Zhang, M. Edirisinghe, Porous Graphene Composite Polymer Fibres, Polymers (Basel). 13 (2020) 76. https://doi.org/10.3390/POLYM13010076.

[2] C. Angulo-Pineda, K. Srirussamee, P. Palma, V.M. Fuenzalida, S.H. Cartmell, H. Palza, Electroactive 3D Printed Scaffolds Based on Percolated Composites of Polycaprolactone with Thermally Reduced Graphene Oxide for Antibacterial and Tissue Engineering Applications, Nanomaterials. 10 (2020) 428. https://doi.org/10.3390/NANO10030428.

[3] N.B. Tolou, H. Salimijazi, M. Kharaziha, G. Faggio, R. Chierchia, N. Lisi, A three-dimensional nerve guide conduit based on graphene foam/polycaprolactone, Mater. Sci. Eng. C - Mater. Biol. Appl. 126 (2021). https://doi.org/10.1016/j.msec.2021.112110.

[4] C. Wan, B. Chen, Poly(ε-caprolactone)/graphene oxide biocomposites: Mechanical properties and bioactivity, Biomed. Mater. 6 (2011) 8. https://doi.org/10.1088/1748-6041/6/5/055010.
